# Cost-effectiveness of endovascular thrombectomy for acute ischemic stroke with established large infarct in Germany: a decision tree and Markov model

**DOI:** 10.1136/jnis-2024-021837

**Published:** 2024-06-21

**Authors:** Sophie Gottschalk, Hans-Helmut König, Fabien Subtil, Susanne Bonekamp, Angelique Denis, Anne Hege Aamodt, Blanca Fuentes, Elke R Gizewski, Michael D Hill, Antonin Krajina, Laurent Pierot, Claus Ziegler Simonsen, Kamil Zeleňák, Martin Bendszus, Götz Thomalla, Judith Dams

**Affiliations:** 1 Department of Health Economics and Health Services Research, University Medical Center Hamburg-Eppendorf, Hamburg, Hamburg, Germany; 2 Hamburg Center for Health Economics, Hamburg, Germany; 3 Service de Biostatistique, Hospices Civils de Lyon, Lyon, France; 4 Laboratoire de Biométrie et Biologie Évolutive, Université Lyon 1, Villeurbanne, France; 5 Department of Neuroradiology, Heidelberg University Hospital, Heidelberg, Germany; 6 Department of Neurology, Oslo University Hospital, Oslo, Norway; 7 The Norwegian University of Science and Technology, Trondheim, Norway; 8 Department of Neurology and Stroke Unit, La Paz University Hospital, Madrid, Spain; 9 Department of Neuroradiology, Medical University of Innsbruck, Innsbruck, Tirol, Austria; 10 Department of Clinical Neurosciences, Hotchkiss Brain Institute, Health Science Centre, University of Calgary & Foothills Medical Centre, Calgary, Alberta, Canada; 11 Department of Radiology, Faculty of Medicine in Hradec Kralove, Charles University, Hradec Kralove, Czech Republic; 12 Department of Neuroradiology, Hôpital Maison-Blanche, Université de Reims Champagne-Ardenne, Reims, France; 13 Department of Neurology, Aarhus University Hospital, Aarhus, Denmark; 14 Clinic of Radiology, Jessenius Faculty of Medicine, Comenius University, Martin, Slovakia; 15 Department of Neurology, University Medical Center Hamburg-Eppendorf, Hamburg, Hamburg, Germany

**Keywords:** Economics, Stroke, Thrombectomy

## Abstract

**Background:**

Recent studies, including the TENSION trial, support the use of endovascular thrombectomy (EVT) in acute ischemic stroke with large infarct (Alberta Stroke Program Early Computed Tomography Score (ASPECTS) 3–5).

**Objective:**

To evaluate the cost-effectiveness of EVT compared with best medical care (BMC) alone in this population from a German healthcare payer perspective.

**Methods:**

A short-term decision tree and a long-term Markov model (lifetime horizon) were used to compare healthcare costs and quality-adjusted life years (QALYs) between EVT and BMC. The effectiveness of EVT was reflected by the 90-day modified Rankin Scale (mRS) outcome from the TENSION trial. QALYs were based on published mRS-specific health utilities (EQ-5D-3L indices). Long-term healthcare costs were calculated based on insurance data. Costs (reported in 2022 euros) and QALYs were discounted by 3% annually. Cost-effectiveness was assessed using incremental cost-effectiveness ratios (ICERs). Deterministic and probabilistic sensitivity analyses were performed to account for parameter uncertainties.

**Results:**

Compared with BMC, EVT yielded higher lifetime incremental costs (€24 257) and effects (1.41 QALYs), resulting in an ICER of €17 158/QALY. The results were robust to parameter variation in sensitivity analyses (eg, 95% probability of cost-effectiveness was achieved at a willingness to pay of >€22 000/QALY). Subgroup analyses indicated that EVT was cost-effective for all ASPECTS subgroups.

**Conclusions:**

EVT for acute ischemic stroke with established large infarct is likely to be cost-effective compared with BMC, assuming that an additional investment of €17 158/QALY is deemed acceptable by the healthcare payer.

**WHAT IS ALREADY KNOWN ON THIS TOPIC**

- Recent randomized controlled trials have demonstrated the benefit and safety of endovascular thrombectomy (EVT) in acute ischemic stroke with large infarct. Previous studies—predominantly conducted for a US and Chinese setting or relying on clinical efficacy data from Asia—indicated that EVT is also cost-effective in this population. The TENSION study, in contrast to previous randomized controlled trials, was conducted in a primarily European population, and inclusion of patients was predominantly based on non-contrast CT.

**WHAT THIS STUDY ADDS**

- The higher upfront costs combined with a significant survival benefit of the EVT group led to higher incremental lifetime costs, but also to higher lifetime quality-adjusted life-years, resulting in a cost-effectiveness ratio that can be considered favorable.

**HOW THIS STUDY MIGHT AFFECT RESEARCH, PRACTICE OR POLICY**

- The results contribute to the evidence that extending the indication for EVT to infarct sizes with an Alberta Stroke Program Early CT Score 3–5 (determined by non-contrast CT) seems economically justified.

## Introduction

 According to current guidelines for the management of acute ischemic stroke, endovascular thrombectomy (EVT) in addition to best medical care (BMC) is indicated in patients with small to medium-sized infarcts (ie, Alberta Stroke Program Early CT Score (ASPECTS) ≥6).^1 2^ Recent randomized controlled trials and a meta-analysis found evidence for superiority of EVT over BMC alone in patients with acute ischemic stroke with a large infarct core (ASPECTS<6), indicated by a better 90-day functional outcome on the modified Rankin Scale (mRS).^3^,^7^

From an economic perspective, a potential change in treatment standards in response to this new evidence (eg, towards an extension of EVT to large infarcts) is likely to increase healthcare costs. As financial resources (eg, healthcare budgets) are not unlimited, it is important to examine whether the clinical benefit can be achieved at an acceptable (additional) cost. Reducing a highly dependent outcome (ie, mRS score 4–5) is particular important in this context, since these patients incur the highest costs for the healthcare system and the society.^8^

Previous studies modeling the cost-effectiveness of EVT in populations with large ischemic infarct have predominantly been conducted from an US healthcare payer or societal perspective and relied on clinical efficacy data from a registry, a prospective cohort study, or the RESCUE-Japan LIMIT trial.^9^,^12^ Another study adopted a Chinese healthcare payer perspective and used clinical input data from the ANGEL-ASPECT trial.^13^ These previous studies found EVT to be cost-effective, meaning that the benefits of EVT can be achieved at an additional cost, but within commonly accepted thresholds. Owing to differences in patient populations and healthcare systems (eg, treatment protocols and reimbursement schemes) between countries and regions, the generalizability of these results to other countries/settings might be limited. A recent study by Moreu *et al* modeled the cost-effectiveness of EVT for several European countries (including Germany), again using efficacy data from the RESCUE-Japan LIMIT trial.^14^ They found varying cost-effectiveness ratios between countries, but overall, they were even more favourable than in the US setting. However, a cost-effectiveness analysis drawing on European input data is still lacking. The TENSION study primarily enrolled patients from European countries (>50% from Germany) and, unlike previous trials that used multimodal brain imaging, inclusion of patients with an ASPECTS of 3–5 was predominantly based on non-contrast CT, which is more likely to reflect clinical practice.^15^ Besides a better outcome after EVT, the TENSION trial is also the first study to find a survival benefit in the EVT group, which might also have economic implications.

Therefore, the aim of this study was to evaluate the cost-effectiveness of EVT compared with BMC alone in patients with acute ischemic stroke with large infarct (ASPECTS 3–5) from a German healthcare payer perspective.

## Methods

This report has been prepared in accordance with the Consolidated Health Economic Evaluation Reporting Standards (CHEERS) and good practice guidelines in decision-analytic modeling.^16 17^ The analysis was a prespecified endpoint of the study protocol, and a health economic analysis plan was developed and submitted to the funder as part of the grant agreement (online supplemental material).

### Study design and model structure

A decision-analytic model to examine the cost-effectiveness of EVT plus BMC versus BMC alone in acute ischemic stroke patients with a large infarct core (ASPECTS 3–5) was built in TreeAge Pro 2023 (TreeAge Software, Williamstown, Massachusetts, USA). The cost perspective adopted was that of a German healthcare payer, who comprehensively covers medical costs (eg, outpatient and inpatient services, medications, etc). Effectiveness was measured by quality-adjusted life-years (QALYs). Cost-effectiveness was expressed by the incremental cost-effectiveness ratio (ICER).

The model consisted of a short-term decision tree representing a 90-day acute phase after the qualifying stroke event and a long-term Markov model (lifetime horizon) (online supplemental figure 1). For both treatment strategies, health states were defined by the mRS, representing the degree of disability at 90 days post-stroke: ranging from 0/1 (no significant disability) to 5 (severe disability), and 6 (death). Patients were assumed to be at no risk of a recurrent stroke in the 90-day acute phase (the number of new ischemic strokes was not significantly different between groups in TENSION).^6^ The risk of intracranial hemorrhage, which was previously found to be higher in the EVT group,^3 4^ was not explicitly modeled as no significant increase in risk was found in the TENSION trial.^6^ After the acute phase, patients entered the Markov model. During each 1-year cycle, the patients could survive or die. In cases of survival, they may remain in their current health state or experience another stroke (potentially leading to further deterioration in health). Patients experiencing a recurrent stroke were assumed to receive BMC as treatment strategy and go through another 90-day acute phase. The health state 90 days after a recurrent stroke determined the health state on entering a new Markov cycle. Costs and health utilities were modeled for each treatment strategy and health state. For the patients transitioning to death or experiencing a recurrent stroke within a Markov cycle, costs and utilities were half-cycle corrected.

Data used in this study were either drawn from published sources, claims data (for the calculation of long-term costs), or the TENSION trial (ClinicalTrials.gov; NCT03094715). The TENSION trial was approved by institutional review boards of all participating study sites and supervised by an independent data monitoring and safety committee, ethics and scientific advisory board.^18^ Written informed consent was obtained from all 253 randomized participants (EVT: n=128; BMC: n=125) or their legal representatives. Further information on demographic and clinical characteristics of the TENSION sample can be found in the main publication.^6^

### Model inputs

Table 1 provides an overview of input parameters. The starting age in the base case model was 72 years (mean age in TENSION) and the distribution of patients across the mRS (transition probabilities) at the beginning of the Markov model corresponded to the 90-day mRS outcome, reflecting the efficacy of EVT.^6^ The stroke recurrence rate by year after qualifying stroke was calculated based on a recent meta-analysis.^19^ The mortality risk was modeled using age-specific mortality rates from German life tables and multiplying these by the relative risk of dying in the respective mRS state.^20 21^

**Table 1:** Model input parameters

Model input parameter	Base case	Range for deterministic sensitivity analysis	Distributional parameters for probabilistic sensitivity analysis*	Source
**Mean age at index stroke (years)**	72	65–80 (IQR)		TENSION trial^6^
**Transition probabilities (90 days post-stroke**)				TENSION trial^6^
EVT				
mRS score 0–1	0.08	0.06 to 0.10 (95% CI)	Beta: 50.92, 573.08	
mRS score 2	0.09	0.07 to 0.11 (95% CI)	Beta: 55.91, 568.09	
mRS score 3	0.15	0.12 to 0.17 (95% CI)	Beta: 90.85, 533.15	
mRS score 4	0.19	0.16 to 0.22 (95% CI)	Beta: 119.81, 504.19	
mRS score 5	0.12	0.10 to 0.15 (95% CI)	Beta: 76.88, 547.12	
mRS score 6 (dead)	0.37	0.33 to 0.41 (95% CI)	Beta: 229.63, 394.37	
BMC				
mRS score 0–1	0.02	0.01 to 0.03 (95% CI)	Beta: 9.98, 629.02	
mRS score 2	0.02	0.01 to 0.03 (95% CI)	Beta: 9.98, 629.02	
mRS score 3	0.12	0.09 to 0.14 (95% CI)	Beta: 73.88, 565.12	
mRS score 4	0.14	0.11 to 0.16 (95% CI)	Beta: 86.86, 552.14	
mRS score 5	0.20	0.16 to 0.23 (95% CI)	Beta: 124.80, 514.20	
mRS score 6 (dead)	0.52	0.48 to 0.56 (95% CI)	Beta: 333.48, 305.52	
**Transition probabilities (after recurrent stroke**)				Goyal *et al*^30^
mRS score 0–1	0.13			
mRS score 2	0.14			
mRS score 3	0.15			
mRS score 4	0.25			
mRS score 5	0.14			
mRS score 6 (dead)	0.19			
**Annual age-specific mortality for Germany**				Federal Statistical Office Germany^20^
**RR of death by mRS score**				Slot *et al*^21^
mRS score 0–1	1			
mRS score 2	1.12	0.82 to 1.56 (95% CI)	Lognormal: 0.11, 0.16	
mRS score 3	1.66	1.24 to 2.23 (95% CI)	Lognormal: 0.51, 0.15	
mRS score 4	1.92	1.41 to 2.61 (95% CI)	Lognormal: 0.65, 0.16	
mRS score 5	2.57	1.92 to 3.43 (95% CI)	Lognormal: 0.94, 0.15	
**Probability of experiencing a recurrent stroke**				Lin *et al*^19^
First year	0.11			
Second year	0.08			
Third year	0.02			
Fourth to fifth year	0.01			
Sixth year onwards	0.02			
**Utilities**				Ali *et al*^23^
mRS score 0–1	0.90	0.89 to 0.91 (95% CI)	Beta: 3577.98, 397.55	
mRS score 2	0.83	0.82 to 0.84 (95% CI)	Beta: 2287.21, 468.46	
mRS score 3	0.68	0.66 to 0.70 (95% CI)	Beta: 1870.60, 880.28	
mRS score 4	0.38	0.36 to 0.40 (95% CI)	Beta: 1012.80, 1652.46	
mRS score 5	0.09	0.07 to 0.11 (95% CI)	Beta: 76.35, 771.98	
mRS score 6 (dead)	0			
**Costs**				
90-Day acute phase				Based on the DRG revenue (G-DRG catalog 2022) and estimated costs depending on documented discharge location and living situation collected in the TENSION trial†
EVT – mRS score 0–5	30 490	(±50%)		
EVT – mRS score 6	15 466	(±50%)		
BMC – mRS score 0–5	25 352	(±50%)		
BMC – mRS score 6	8814	(±50%)		
Recurrent stroke	16 670	(±50%)		
Annual long-term costs				BARMER health insurance data
mRS score 0;1 (CD 0)	6970	6753 to 7187 (95% CI)	Gamma: 3976.22, 0.57	
mRS score 2 (CD 1)	7019	6464 to 7575 (95% CI)	Gamma: 613.20, 0.09	
mRS score 3 (CD 3)	18 917	17 900 to 19 934 (95% CI)	Gamma: 1329.08, 0.07	
mRS score 4 (CD 4)	19 539	18 288 to 20 790 (95% CI)	Gamma: 937.21, 0.05	
mRS score 5 (CD 5)	19 315	17 221 to 21 409 (95% CI)	Gamma: 326.99, 0.02	

* Beta distribution: alpha, beta; Lognormal distribution: log of mean, standard error; Gamma distribution: alpha, lambda.

† See supplemental material for a detailed description

BMC, best medical care; CD, care degree; CI, confidence interval; EVT, endovascular thrombectomy; IQR, interquartile range; mRS, modified Rankin Scale; RR, relative risk.

Costs were modeled for each treatment strategy and health state. Acute care costs for the EVT and BMC strategies were calculated from the diagnosis-related group (DRG) revenue (G-DRG catalog 2022) and based on the average number of nights in hospital for treatment of the qualifying stroke event in TENSION (15 nights in both groups). In the G-DRG system, cases are grouped in a specific DRG, which is mainly determined by the diagnosis (ICD-10) and provided operations and procedures. Each DRG is assigned a weight, which is multiplied by the base rate of a respective cost year (reflecting the amount of services needed for an average hospital case). Within defined DRG-specific boundaries regarding the length of stay, a case is reimbursed by a flat rate. When crossing the upper or lower boundaries, surcharges or deductions apply. These boundaries for the length of stay do not apply for in-hospital nursing care, which is reimbursed according to the actual length of stay by a daily fee multiplied by the DRG-specific nursing cost weight. In the absence of detailed documentation of diagnoses (ICD-10 codes) and procedures (operation and procedure codes) for each TENSION participant, the most likely DRG was assumed for the EVT (DRG B39B) and BMC (DRG B70B) groups, based on ischemic stroke (ICD-10 I63.3) as the assumed main diagnosis and the operation and procedure codes for complex neurological stroke treatment (8–981.3; both groups) and thrombectomy (8–836.80; EVT group only). For the EVT strategy, additional remuneration for the material needed for the EVT (ie, aspiration catheters and stent retrievers) was considered. Furthermore, trial information on the discharge location after treatment of the qualifying stroke event (ie, home, assisted living, nursing home, rehabilitation, hospital) and the living situation at 90-days' follow-up were used to estimate patient pathways in the first 90 days and derive average costs (online supplemental figure 2). The total average acute costs were calculated from the estimated costs for each pathway, considering the proportion of patients in each pathway. A detailed description of the assumptions and the calculation of acute costs is provided in the supplemental material (online supplemental tables 1–3).

Long-term healthcare costs, which included outpatient and inpatient services, pharmaceuticals, sickness benefits, medical aids, and cost for formal care and rehabilitation, were based on 2022 routine data from a large German health insurance company (BARMER) for patients diagnosed with ICD-10 code I63.3. To calculate mRS-specific costs, mRS states were matched to German care degrees (0 to 5), which qualify for different nursing care insurance benefits depending on the level of impairment in everyday life; no care degree was assumed for mRS score 0–1, mRS score 2 was assigned care degree 1, and mRS scores 3–5 were assigned to care degrees 3–5, respectively.^22^ People with care degree 2 were not assigned to a specific mRS score and were therefore excluded from the analyses. In addition, to ensuring a consistent mRS classification, only people with no change in care degree in 2022 were included. Age-specific mean annual costs were calculated for each care degree based on a regression model that included dummies for mRS conditions and age as independent variables. All costs were reported in 2022 euros. German EQ-5D-3L utility values were assigned to the mRS states and QALYs were calculated by multiplying the mRS-specific utility by the time spent in the respective health state.^23^ Costs and QALYs were discounted by an annual 3% rate.^24^

### Base case and subgroup analysis

In the base case analysis, cost-effectiveness was determined using a cohort simulation with the most likely parameter estimates. The ICER was calculated as the difference in mean costs divided by the difference in mean effects between the EVT and BMC group:


$$ICER=\frac{\overset{-}{Costs_{EVT}}-\;\overset{-}{Costs_{BMC}}}{\overset{-}{QALYs_{EVT}}-\;\overset{-}{QALYs_{BMC}}}$$

In the absence of an official cost-effectiveness threshold in Germany, a recently proposed threshold based on the increase in life expectancy and healthcare expenditure has been used, which for Germany is 1.01 times the per capita gross domestic product (€46 264 in 2022).^25 26^ Hence, an ICER below €46 727 per QALY was considered cost-effective in this study.

In a subgroup analysis, the cost-effectiveness of EVT was analyzed separately for populations with an initial ASPECTS of 3, 4, or 5. To this end, the proportions of patients in 90-day mRS states were altered according to the distribution of TENSION participants in the respective ASPECTS (online supplemental table 4). The model structure and other input data remained identical to those of the base case analysis.

### Sensitivity analysis

Deterministic one-way sensitivity analyses were performed to examine the influence of individual input parameters on the ICER by varying the parameters over a range of values (table 1). The robustness of the base case results was further assessed in a probabilistic sensitivity analysis, using a Monte Carlo simulation to simultaneously vary input parameters. Across 10 000 iterations, parameter values were sampled according to distributional assumptions (gamma distribution for costs, beta distribution for probabilities or utilities, and lognormal distribution for relative risks; table 1). The results were visualized by a cost-effectiveness acceptability curve, showing the probability of cost-effectiveness as a function of the willingness to pay for an additional QALY.

### Model validation

The face validity of the model structure, input values, assumptions, as well as the plausibility of the results were judged by clinical experts (neurologists of the TENSION trial). Internal validity was examined by varying the input parameters and checking whether the results would change in the hypothesized direction—for example, reducing the proportion of patients with mRS score 6 at 90 days (ie, using the efficacy input from the RESCUE-Japan LIMIT trial) decreased the difference in lifetime costs and QALYs between EVT and BMC. The model was further cross-validated by comparing the results with those of previous, related studies and examining the reasons for differences in the results (see Discussion section).

## Results

Base case and subgroup analysis results are presented in table 2. EVT compared with BMC had higher lifetime costs (€110 952 vs €86 695) and higher lifetime effects (2.84 vs 1.42 QALYs). The resulting ICER was €17 158 per QALY gained. Looking at ASPECTS 3–5 separately, the lowest incremental costs (€16 328), but also the lowest incremental QALYs (0.63), were incurred by patients with an ASPECTS of 3. The highest incremental costs (€31 537) and QALYs (2.06) were incurred by patients with an ASPECTS of 5. Consequently, the ICER was highest in patients with an ASPECTS of 3 (€25 939/QALY) compared with those with an ASPECTS of 4 and 5 (€15 164/QALY and €15 321/QALY, respectively).

**Table 2:** Costs (in 2022 euros), QALYs, and ICERs for EVT versus BMC

	EVT	BMC	Δ
**ASPECTS 3–5 (base case**)			
Costs (€)	110 952	86 695	24 257
QALYs	2.84	1.42	1.41
ICER (€/QALY)			17 158
**ASPECTS 3**			
Costs (€)	99 395	83 068	16 328
QALYs	2.10	1.47	0.63
ICER (€/QALY)			25 939
**ASPECTS 4**			
Costs (€)	114 231	92 398	21 833
QALYs	2.71	1.27	1.44
ICER (€/QALY)			15 164
**ASPECTS 5**			
Costs (€)	117 051	85 514	31 537
QALYs	3.56	1.50	2.06
ICER (€/QALY)			15 321

ASPECTS, Alberta Stroke Program Early CT Score; BMC, best medical care; EVT, endovascular thrombectomy; ICER, incremental cost-effectiveness ratio; QALYs, quality-adjusted life-years; Δ, delta (difference between EVT and BMC).

**Figure 1:**
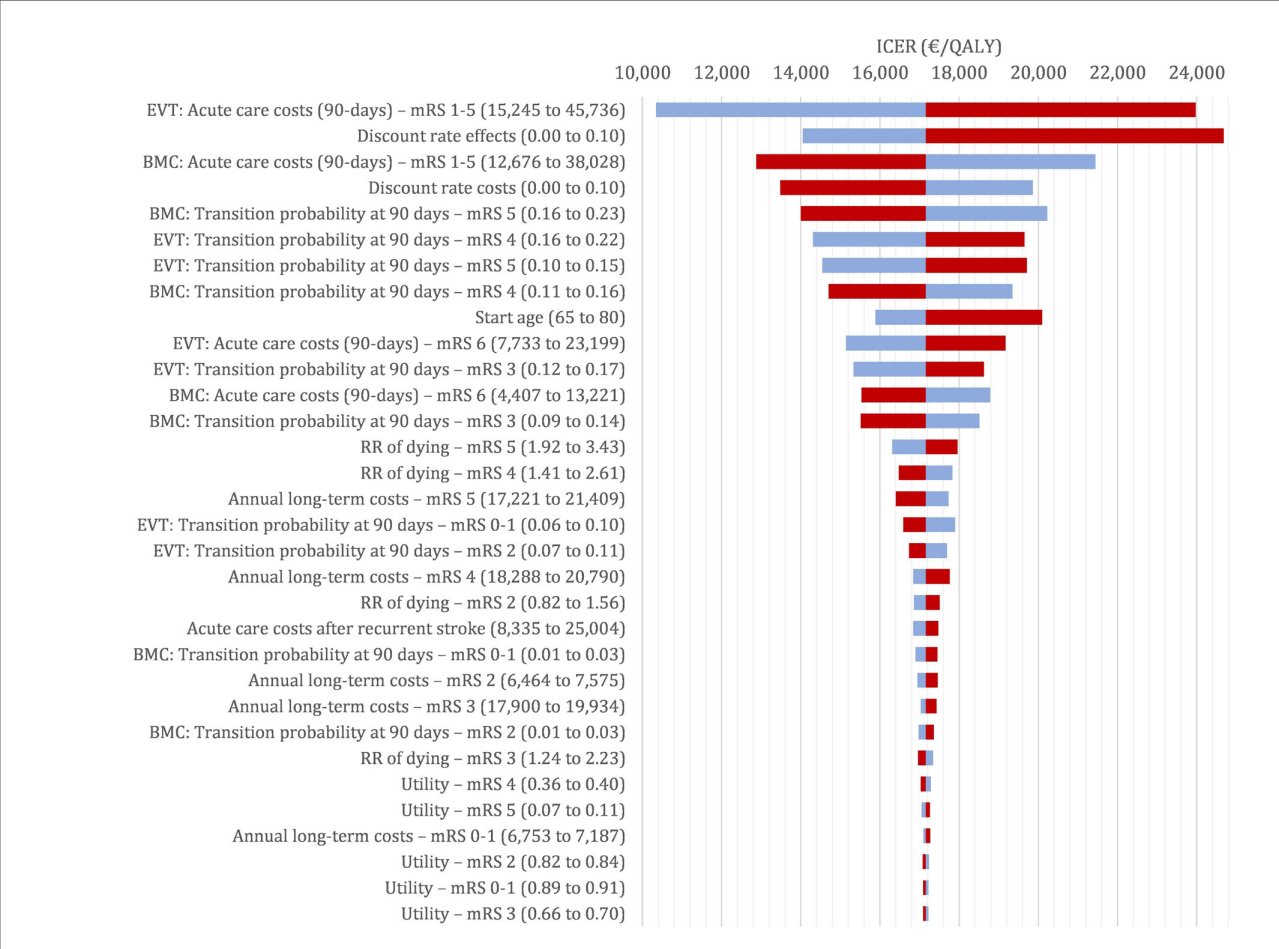
Tornado diagram presenting the results of the deterministic sensitivity analysis. Blue bars: effect of decreasing the parameter value, red bars: effect of increasing the parameter value. BMC, best medical care; EVT, endovascular thrombectomy; ICER, incremental cost-effectiveness ratio; mRS, modified Rankin Scale; QALY, quality-adjusted life-year.

**Figure 2:**
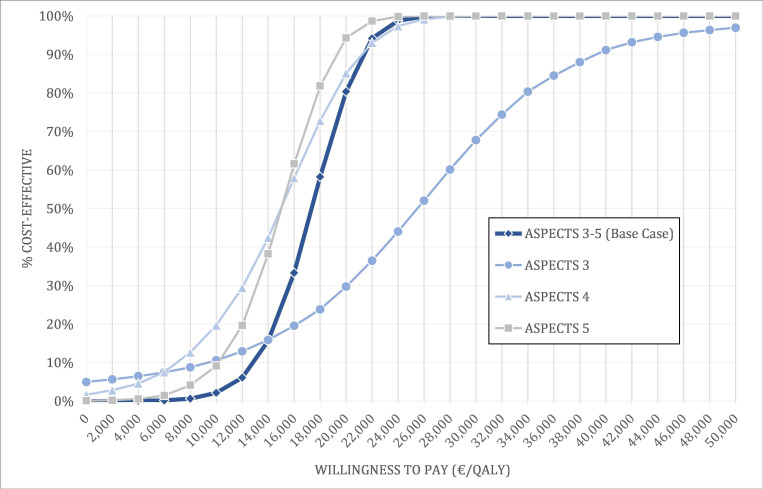
Cost-effectiveness acceptability curves presenting the results of the probabilistic sensitivity analyses. Probability of cost-effectiveness depending on the willingness to pay. ASPECTS, Alberta Stroke Program Early CT Score; QALY, quality-adjusted life-year.

In the deterministic sensitivity analysis, the ICER was most sensitive to acute care costs for the EVT strategy, followed by the discount rate for the effects, acute care costs for the BMC strategy, the discount rate for costs, the proportion of patients with mRS score 4 or 5 after 90 days, and age (figure 1). However, the ICER was below €25 000 per QALY for all parameter variations, suggesting that EVT would still be considered cost-effective. In the probabilistic sensitivity analysis, the probability of cost-effectiveness reached 95% at a willingness to pay >€22 000 per QALY (figure 2). In the ASPECTS 3 subgroup, 95% probability of cost-effectiveness was only achieved at a willingness to pay of €44 000 per QALY. The results for the ASPECTS 4 and 5 subgroups were close to the base case results.

## Discussion

In the current study, EVT for patients with large ischemic infarct (ASPECTS 3–5) was cost-effective compared with BMC alone from a German healthcare payer perspective and over a lifetime horizon. The ICER point estimate was €17 158, which can be considered an acceptable investment under recent threshold recommendations.^25^ The results were robust to parameter variation in sensitivity analyses (eg, in the probabilistic sensitivity analysis, high certainty of cost-effectiveness was achieved at a willingness to pay of >€22 000 per QALY).

The results of our study are in line with the general consensus of previous studies examining the cost-effectiveness of EVT in populations with large ischemic infarcts that the benefits of EVT can only be achieved at an additional, but justifiable cost.^11^,^14^ The current study contributes to this field by using data on the efficacy of EVT in a population with large ischemic infarcts from the TENSION study, which demonstrated the benefits of EVT in a predominantly European population,^6^ whereas previous clinical trials were conducted in Asia or the USA. Moreover, the TENSION study included patients based on imaging procedures commonly used in clinical practice (eg, unenhanced CT) and found a shift towards a better 90-day functional outcome on the mRS scale, and also a survival benefit. This survival benefit was less pronounced in the RESCUE-Japan LIMIT trial, which was used to populate previous cost-effectiveness models in the USA and Europe.^11 12 14^

Even though the ICER was in a similar range to that found by Sanmartin *et al* (US$16,239/QALY)^11^ and Ospel *et al* (US$15,743/QALY),^12^ the incremental QALYs and incremental costs were higher in the current study, reflecting the survival difference between EVT and BMC in the TENSION trial. Overall, a higher proportion of patients in the EVT and BMC group had died after 90 days in TENSION than in RESCUE-Japan LIMIT. In the latter, the largest between-group difference in favor of EVT was observed in patients with mRS score 5, a population with high care needs and hence high costs.^3^ A lower proportion of patients with mRS score 5 in the EVT group compared with the BMC group (in RESCUE-Japan LIMIT) thus has a favorable effect on costs in the EVT group, while a higher proportion of survivors in the EVT group (in TENSION) is inherently associated with higher costs. However, a survival benefit also means additional lifetime and thus a gain in QALYs, which are accumulated and factored into the ICER equation as denominator. This is why, despite the significantly higher costs in the EVT group, the ICER in the current study was in a range that can be regarded as good value for money.

The ICER in the current study was higher than the one reported by Moreu *et al* for Germany (€17 158 vs €3933 per QALY),^14^ indicating potentially higher budgetary impacts for the healthcare payer. Besides the fact that the efficacy of EVT used in the model by Moreu *et al* was also based on the RESCUE-Japan LIMIT trial, long-term costs were taken from a previous German thrombolysis study that only considered care-related costs,^22^ and QALYs were calculated using UK-specific utility values. Our analysis goes beyond the study by Moreu *et al* by using primarily European data on the efficacy of EVT, German-specific utilities to calculate QALYs, and long-term costs including all cost categories covered by the statutory health and long-term care insurance. Despite the differences in methods and results between Moreu *et al* and the current study, the conclusion remains that EVT for acute ischemic stroke with established large infarct is cost-effective from a German healthcare payer perspective.

In the previous cost-effectiveness analysis of EVT for patients with a large core stroke by Ospel *et al*, EVT was cost-effective in patients with ASPECTS 4–5 but not in those with ASPECTS 3 from an US healthcare payer and societal perspective.^12^ In our analysis using efficacy data from the European TENSION trial and adopting a German healthcare payer perspective, we could not replicate this finding. In contrast, EVT was cost-effective for all ASPECTS subgroups, including the subgroup with an ASPECTS 3: Although the ICER was less favorable than for the subgroups with an ASPECTS 4 or 5 (€25 939 vs €15 164/€15 321, and with a 95% probability of cost-effectiveness at a willingness to pay of €44 000), EVT can still be considered cost-effective for patients with an ASPECTS of 3.^25^

Patients with an ischemic stroke with large vessel occlusion and extensive infarction are among the most severely affected and vulnerable stroke patients with a dismal prognosis. Given the severity of the disease, health economic analyses of treatment approaches are of particular interest. In recent randomized trials of EVT for large-vessel stroke, the mortality rate and the proportion of patients who were severely disabled at 90 days were high even in these patients, although significantly lower than with medical treatment alone. Against this background, evidence of cost-effectiveness is important and helpful information that can support treatment recommendations for clinical practice.

Some limitations of our study should be mentioned. First, like any model, the decision-analytic model is a simplification of reality that does not take into account, for example, individual-level differences in (acute) costs and QALYs, which might vary depending on additional adverse events, comorbidities, and individualized treatments. Although frequently applied to evaluate cost-effectiveness in the context of stroke care, the Markov modeling technique is subject to some limitations: for example, the fixed cycle length, which might not be sufficient to represent transitions/disease trajectories within shorter time periods, and the Markov assumption, that is, the probability of transitioning to a particular health state is independent of previous transitions. Alternative modeling techniques such as discrete event simulations might allow a more accurate representation of individual disease trajectories, taking into account individual patient characteristics and event trajectories in predicting the occurrence and timing of future events (and thus improve model validity).^27 28^ Also, while discounting future costs and effects to account for time preference, the model did not account for potential future changes in treatment strategies, reimbursement schemes, care reforms, etc that could affect the cost-effectiveness.

Second, the initial transition probabilities were based on the 90-day mRS outcome in the TENSION trial, and the model structure did not allow for improvement on the mRS beyond these 90 days, in contrast to studies that found such an improvement to be possible.^29^ This might have led to an underestimation of the treatment effect in the model.

Third, the costs assigned to the different health outcomes in the model were limited to the perspective of a healthcare payer, thereby ignoring broader societal costs, such as productivity loss (less relevant in a predominantly retired population) or informal care. Furthermore, the long-term costs were based on a general stroke population and the matching of care degrees to mRS scores was based on expert rating.^22^

Fourth, caution is warranted in interpreting the subgroup analyses, as the TENSION trial was not powered for an analysis of the 90-day mRS outcome by ASPECTS and was stopped early for efficacy, further reducing the sample size.

Finally, the generalizability of the results to other healthcare systems is limited, as the model was developed to evaluate the cost-effectiveness of EVT for large ischemic infarcts in the German healthcare system.

### Conclusion

EVT for acute ischemic stroke with established large infarct (ASPECTS 3–5) is likely to be cost-effective compared with BMC in Germany, assuming that an additional investment of €17 158 per QALY is deemed acceptable by the healthcare payer. The results contribute to the evidence that extending the indication for EVT to infarct sizes of ASPECTS 3–5 is of clinical benefit and seems economically justified.

## Supplementary material

10.1136/jnis-2024-021837 online supplemental file 1

## Data Availability

All data relevant to the study are included in the article or uploaded as supplementary information.
