# Supplementary material for: Cost-effectiveness of endovascular thrombectomy for acute ischemic stroke with established large infarct in Germany: a decision tree and Markov model
Source: J Neurointerv Surg. 2024 Jun 21;17(7):e021837. doi: 10.1136/jnis-2024-021837 (PMC12322465; doi:10.1136/jnis-2024-021837)
Supplement: online supplemental file 1 [file jnis-17-7-s001.pdf]

Supplemental material

Contents

Model structure 2

Model input 3

Calculation of acute care costs 3

Transition probabilities (90-day mRS outcome) by ASPECTS 7

Health economic analysis plan 8

Model structure

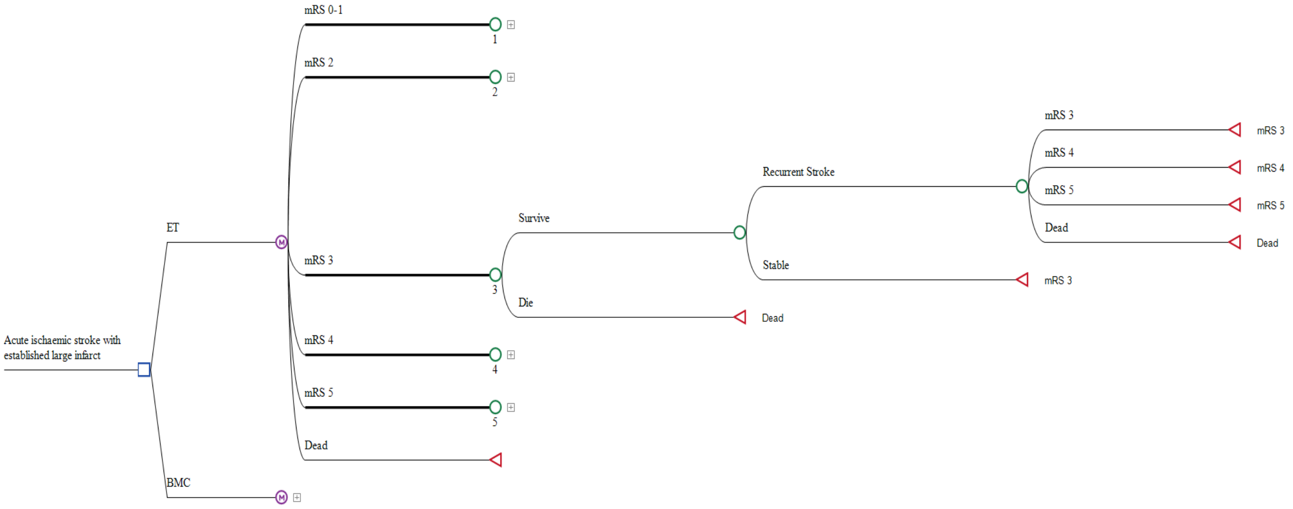

Figure S1 Model structure. BMC, best medical care; ET, endovascular thrombectomy; mRS, modified Rankin Scale

Model input

Calculation of acute care costs

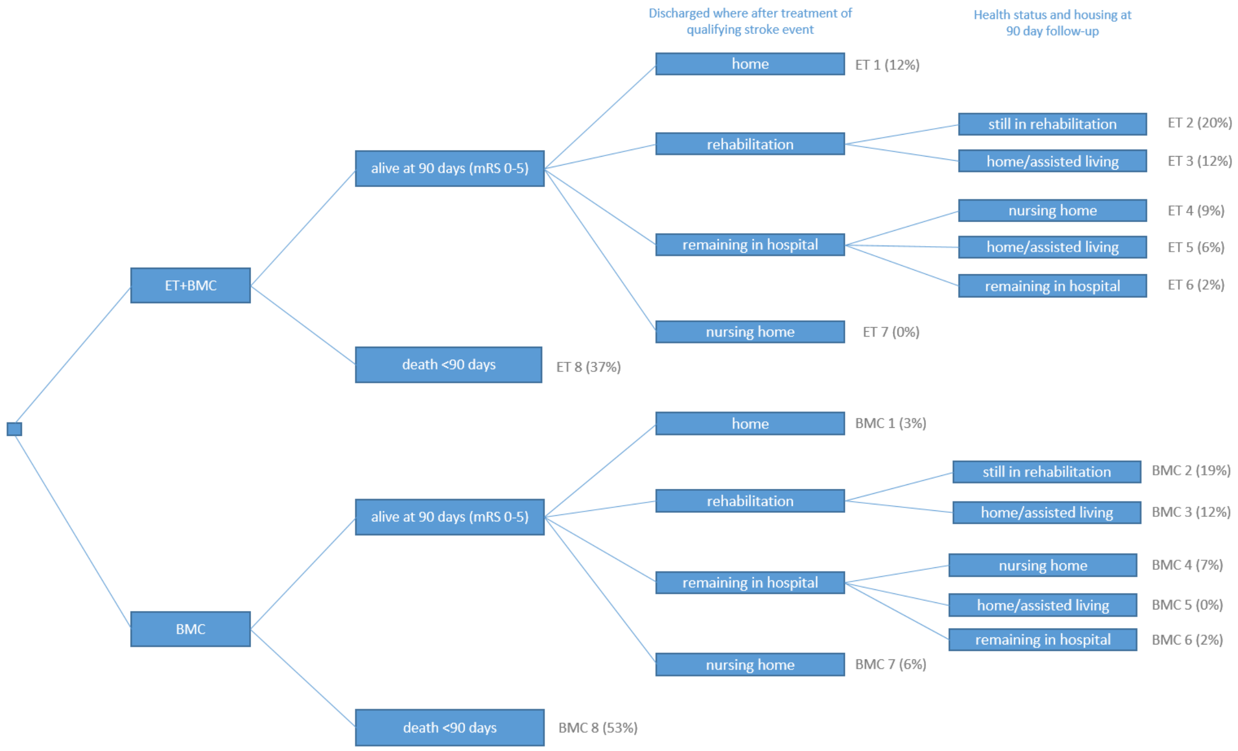

Figure S2 Patient pathways within 90 days after the qualifying stroke event. Based on TENSION trial data (unpublished).

Table S1 Assumptions and values used to calculate costs of the index hospitalisation

	**EVT**	**BMC**
Diagnosis (ICD-10 code)	I63.3<br>Cerebral infarction due to thrombosis of cerebral arteries	
Procedures (OPS code; description freely translated)	8-981.33<br>Complex neurological treatment of acute stroke; on a stroke unit with the possibility of performing thrombectomies and intracranial interventions; more than 96 hours.	
	8-836.80<br>Thrombectomy: Intracranial vessels	
	(8-98f.0)†<br>Complex intensive care treatment (basic procedure)	

DRG<br>(based on G-DRG catalogue 2022; description freely translated)	B39B<br>Neurological complex treatment of acute stroke with specific OR procedure, up to 72 hours with complex intervention or more than 72 hours, without complicated procedure, without complicated constellation, without complex intensive care treatment > 392 / 368 / - points.	B70B<br>Apoplexy with complex neurological treatment of acute stroke, more than 72 hours, without complicating diagnosis or with complex cerebrovascular vasospasm or intensive care complex treatment > 196 / 184 / - effort points.
DRG base rate 2022	€3,833.07	
Nursing care fee 2022 (per day)	€200	
Average number of days in hospital, 90-day mRS 0-5*	15	15
Average number of days in hospital, 90-day mRS 6*	11	12
DRG revenue<br>(without surcharges or deductions and nursing care fee)	€10,038.81<br><br>DRG B39B (BWR=2.619)	€5,285.80<br><br>B70B (BWR=1.379)
Total DRG revenue (including nursing care fee) – 90-day mRS 0-5	€14,774.01	€9,696.40
Total DRG revenue (including nursing care fee) – 90-day mRS 6	€13,511.29	€8,814.28
Additional fees stent retriever<br><br>(based on G-DRG catalogue 2022)	€1,682.24	n.a.
Additional fees aspiration catheter<br><br>(hospital-specific fee for the University Medical Center Hamburg-Eppendorf in 2022)	€800	n.a.
Average additional fees for EVT materials ǂ	€1,954.44	n.a.
**Total cost of index hospitalisation – 90-day mRS 0-5**	**€16,728.45**	**€9,696.40**

**Total cost of index hospitalisation – 90-day mRS 6**	**€15,465.73**	**€8,814.28**
* based on TENSION trial data (unpublished)<br>† 54% (EVT) vs. 29% (BMC) of the patients were treated on the intensive care unit. According to the 2022 documentation from the Medical Controlling of the University Medical Center Hamburg-Eppendorf, 8-98f.0 was the most frequently coded intensive care OPS for people who also received complex neurological treatment for acute stroke. Including this OPS did not change the DRG grouping.<br>ǂ In the EVT group, a stent retriever was used in 17.4%, an aspiration catheter in 23.1%, and both in 59.5%<br><br>Notes: BWR, Bewertungsrelation (relative cost weight, which is multiplied with the DRG base rate); DRG, diagnosis-related group(s); EVT, endovascular thrombectomy; ICD-10, International Statistical Classification of Diseases and Related Health Problems, tenth Revision; mRS, modified Rankin scale; OPS, Operationen- und Prozedurenschlüssel (operation and procedure code)		

Table S2 Further costs between index hospitalisation and 90-day FU by patient pathways (90-day mRS 0-5)

**Patient pathway until 90-day FU**	**Assumptions and costs**	**EVT** (% of patients with a 90-day mRS 0-5)	**BMC** (% of patients with a 90-day mRS 0-5)
1) Discharged home after qualifying stroke event	No additional cost incurred until 90-day FU	19.7	5.4
2) Discharged to rehabilitation after qualifying stroke event; still in rehabilitation at 90-day follow-up	75 days rehabilitation (€203.57/day*)<br><br>→ **€15,267.75**	32.4	39.3
3) Discharged to rehabilitation after qualifying stroke event; discharged home/assisted living after rehabilitation	6 weeks (42 days) rehabilitation (€203.57/day*)<br><br>→ **€8,549.94**	19.7	25.0
4) Remaining in hospital after treatment of qualifying stroke event; living in	15 additional nights in hospital (€1,114.43/day*)	14.1	14.3

nursing home at 90-day follow-up	2 month (60 days) in nursing home (€118.12/day*)<br><br>→ **€23,803.65**		
5) Remaining in hospital after treatment of qualifying stroke event; living at home/assisted living at 90-day follow-up	15 additional nights in hospital (€1,114.43/day*)<br><br>→ **€16,716.45**	9.9	0.0
6) Remaining in hospital after treatment of qualifying stroke event; still in hospital at 90-day follow-up	75 additional nights in hospital (€1,114.43/day*)<br><br>→ **€83,582.25**	2.8	3.6
7) Discharged to nursing home after treatment of qualifying stroke event	75 days in nursing home (€118.12/day*)<br><br>→ **€8,859.00**	0.0	12.5
*standardized unit cost reported in "Muntendorf LK, Konnopka A, Brettschneider C, et al. [Updating standardized unit costs for health economic evaluation from a societal perspective]. *Gesundheitswesen* 2024", inflated to 2022 euros			

Table S3 Total 90-day acute costs, comprising cost of index hospitalisation and further costs, considering the proportion of patients in each pathway (see Table S2 above)

	**EVT**	**BMC**
**Average acute care costs – 90-day mRS 0-5**	**€30,490.43**	**€25,351.74**
**Average acute care costs – 90-day mRS 6**	**€15,465.73**	**€8,814.28**

Transition probabilities (90-day mRS outcome) by ASPECTS

Table S 4 Transition probabilities (90 days post-stroke) by ASPECTS

	ASPECTS 3		ASPECTS 4		ASPECTS 5	
	**Base case**	**Sensitivity analysis**	**Base case**	**Sensitivity analysis**	**Base case**	**Sensitivity analysis**
EVT						
mRS 0-1	0.06	Beta: 10.35, 169.06	0.04	Beta: 10.28, 214.04	0.14	Beta: 35.10, 188.14
mRS 2	0.03	Beta: 5.05, 174.03	0.11	Beta: 27.42, 199.11	0.12	Beta: 28.72, 193.12
mRS 3	0.14	Beta: 28.15, 154.14	0.11	Beta: 27.42, 199.11	0.19	Beta: 48.88, 178.19
mRS 4	0.19	Beta: 41.97, 144.19	0.27	Beta: 79.14, 164.27	0.11	Beta: 27.49, 194.11
mRS 5	0.11	Beta: 21.86, 159.11	0.11	Beta: 27.42, 199.11	0.15	Beta: 36.41, 187.15
mRS 6 (dead)	0.47	Beta: 154.59, 94.47	0.36	Beta: 119.82, 144.36	0.30	Beta: 89.12, 154.30
BMC						
mRS 0-1	0.02	Beta: 5.04, 234.02	0.00	Beta: 0.00, 37.02	0.02	Beta: 5.05, 199.02
mRS 2	0.02	Beta: 5.04, 234.02	0.02	Beta: 4.02, 190.03	0.00	Beta: 1.00, 203.00
mRS 3	0.11	Beta: 29.69, 212.11	0.08	Beta: 17.01, 178.08	0.15	Beta: 35.46, 173.15
mRS 4	0.14	Beta: 37.27, 206.14	0.17	Beta: 38.49, 161.17	0.10	Beta: 22.80, 183.10
mRS 5	0.17	Beta: 46.68, 199.17	0.24	Beta: 59.79, 147.24	0.19	Beta: 45.20, 166.19
mRS 6 (dead)	0.54	Beta: 273.97, 109.54	0.49	Beta: 178.13, 99.49	0.53	Beta: 224.12, 95.53

Notes: ASPECTS, Alberta Stroke Program Early Computed Tomographic Score; BMC, best medical care; CI, confidence interval; mRS, modified Rankin Scale; RR, relative risk.

Health economic analysis plan

Contents

A Brief Overview 4
B The Decision-Analytic Model 6
B.1. Description of the Markov Model 6
B.1.1. Markov Nodes 8
B.1.2. Markov Cycle Subtrees 9
mRS 0-5 9
Death 9
B.2. Outcomes 9
B.2.1. Costs 9
B.2.2. Quality-adjusted life years 10
B.2.3. Incremental cost-effectiveness ratio (ICER) 10
B.3. Data 12
B.3.1. Baseline Cohort 12
B.3.2. mRS scores 12
B.3.3. Mortality Data 12
B.3.4. Treatment Effect 12
B.3.5. Quality-adjusted life years 13
B.3.6. Cost Data 13
Price Conversion 13
Discounting 13
B.4. Statistical Analyses 15
B.4.1. Cost-Effectiveness Analyses 15
B.4.2. Sensitivity Analyses 15
B.4.3. Software 16
C References 17

Tables and Figures

Table 1: Parameter values for Markov model 14

Figure 1. Markov tree of the Markov model. 7

A Brief Overview

International guidelines recommend intravenous thrombolysis with recombinant tissue Plasminogen Activator (rtPA, Alteplase) to treat actue ischemic stroke within the first 4.5 hours. Furthermore, for patients with contraindications for Alteplase (e.g. anticoagulation, recent surgery, time after system onset beyond 4.5 hours) endovascular thrombectomy has been recommended. Previous studies focused on the comparison of endovascular thrombectomy to best medical care (i.e. iv thrombolysis if indicated, supportive care on a dedicated stroke unit) in patients who had exceeded the time window for alteplase but still had good prognostic factors (i.e. short time interval between stroke onset and endovascular treatment as well as small size of ischemic lesion prior to treatment). Studies on the efficacy and safety of endovascular thrombectomy in patients with unfavourable prognostic factors (ASPECT score of 3-5, treatment time window of up to 12 hours from symptom onset or last seen well (if case of unknown time of symptom onset)) have not been published yet. However, especially for patients with unfavourable prognostic factors and inadequate care high follow-up costs, such as costs for long-term care, might occure. Therefore, appropriate treatment for these patients has the potential to reduce long-term costs.

Within the framework of the TENSION trial, we will develop a Markov model to evaluate the long-term economic consequences of endovascular thrombectomy (ET) compared with best medical care (BMC), and to estimate the influence on health-related quality of life (HrQoL). HrQoL will be measured by the EQ-5D-3L. Costs will be measured from the health care perspective of different European countries. Data on ET efficacy will be taken from the TENSION trial. Key assumptions affecting cost and HrQoL will be investigated by univariate and probabilitic sensitivity analyses. Both strategies branches in the model, that is ET and BMC, will have the same structure regarding Markov health stages and Markov cycle sub-trees. Differences will be realized by locally assigning strategy-specific values for probabilities, costs and outcome variables.

In the model, patients will either be treated with BMC or ET and categoriesed by their modified ranking scale (mRS) scores 90 days after stroke into seven Markov states to map patients' disability after stroke from 0 (perfect health) to 6 (dead). Hereafter, patients can die, remaine stable, or suffer from recurrent stroke (RS).

The following outcomes will be included in the model: Quality-adjusted life years (QALYs), costs from a health care perspective, incremental cost-effectiveness ratio (ICER).

B The Decision-Analytic Model

B.1. Description of the Markov Model

A decision-analytic Markov model will be used in which a hypothetical cohort of patients moves through health states based on the mRS scale (see Figure 1).

Technically, the model will compare two competing strategies in patient with a stroke having unfavourable prognostic factors: (1) best medical care (BMC), (2) endovascular thrombectomy (ET).

Transitions from one state to another will be defined by transition probabilities derived from the published literature. The model will discriminate states based on the mRS scores 0-5 and death. mRS scores at 90 days will be assumed to be equivalent to those at 1 year. Conditionally on these Markov states patient will either be stable in their mRS scores, experience a recurrent stroke (RS) or die. Thereby, transitions between mRS scores will be allowed only from low to high mRS scores. Age-specific mortality will be adapted for patients surviving more than one year by the relative risk to die in each mRS state. Furthermore, mortality will be adapted by the relative risk to die from a RS. Costs and utilities will be assigned to each Markov state. Costs will comprise health care utilization for acute care (hospital stays and medication due to a stroke) as well as mRS-specific long-term care costs. mRS-specific utilities will be cacluated based on the EQ-5D-3L and assigned to each Markov-state. An annual cycle length will be used and the simulation is carried out for 25 years.

The model estimates quality-adjusted life years (QALYs) and total costs for each strategy. Incremental cost-effectiveness will be determined in Euro per quality-adjusted life-year gained (€/QALY), respectively. Costs and effects will be discounted according to discount rates recommended by current guidlines. A health care perspective will be chosen.

Figure 1. Markov tree of the Markov model.

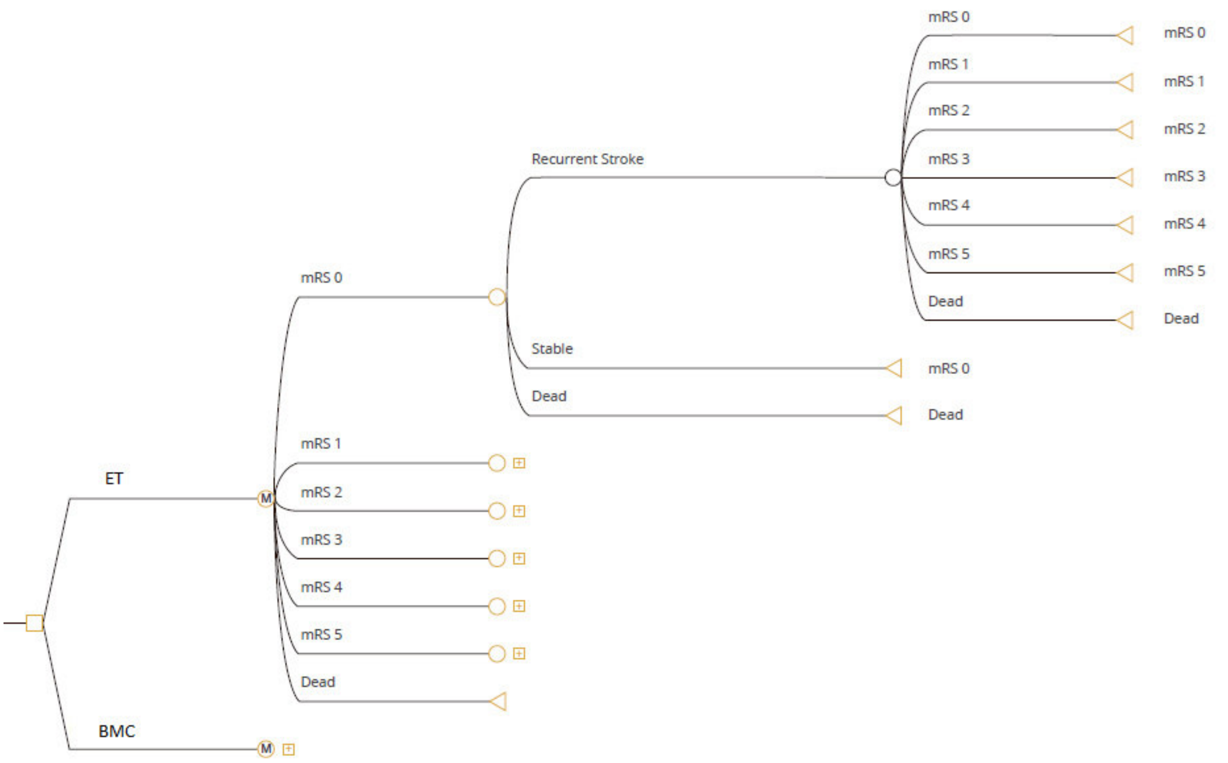

Model structure for ET in patients with unfavourable prognostic factors compared with BMC. Patients will enter the model with ischemic stroke, receiving ET or BMC, moving to a health state defined by the mRS. After treatment, patients will enter annual cycles, in which the patient will remain in the same mRS state unless suffering from recurrent stroke or dying. ET indicates endovascular thrombectomy; BMC best medical care; mRS, modified Ranking Scale.

Decision Branches

The decision node will lead to the competing strategies:

(1) best medical care (BMC), which is the standard comparator,

(2) endovascular thrombectomy (ET).

All strategies will have the same structure concerning Markov health states and subtrees. Differences will be indicated by locally assigned values for probability and outcome variables at each strategy branch.

B.1.1. Markov Nodes

Each decision strategy will lead to a Markov node with 7 different Markov health states:

1. mRS 0
2. mRS 1
3. mRS 2
4. mRS 3
5. mRS 4
6. mRS 5
7. death (= mRS 6; absorging health state)

Age, gender, mRS distribution of the initial population will be determined based on pending data from the TENSION trial.

B.1.2. Markov Cycle Subtrees

mRS 0-5

Disability after stroke will be measured by mRS scores 0-5, which will be included into the model as Markov states. We will assume that patient can die or survive afterwards. Furthermore, patients will be able to stay stable in their mRS score or suffer from a RS and consequently can stay stable or detoriate in their mRS score. For each mRS stage costs and utilities will be evaluated, which will be taken from the data of the TENSION trial.

The Markov cycle subtrees for mRS 1-5 states will be dynamic clones of the Markov cycle subtree for mRS 0.

Death

This health state will contain all patients who died, and will be an absorbing Markov state.

B.2. Outcomes

The following outcomes will be included in the model, and therefore can be evaluated:

- quality-adjusted life years (QALYs),
- costs from a health care perspective,
- incremental cost-effectiveness ratio (ICER).

B.2.1. Costs

Long-term care costs will be determined by matching mRS scores to care degrees based on an expert interview. To match care degrees with mRS scores, two patient vignettes per mRS score based on official training examples for medical staff will be used (Medical University of South Carolina, 2019). Patients with mRS scores 0 or 1 will not qualify for a care degree. Patients with

mRS score 2 will be allocated to care degree 1 (minor impairments), mRS score 3 to care degree 3 (serious impairments), mRS score 4 to care degree 4 (severe impairments), and mRS score 5 to care degree 5 (most severe impairments).

Each participant in the TENSION trial will be assigned a care degree based on the mRS scores at at 90 days and at 12 months after stroke. The costs for long-term care will thus depend on the care degree and the current living situation (nursing home, community-dwelling). The care levels will be monetised with payments from the long-term care insurance for the year 2022.

B.2.2. Quality-adjusted life years

QALY will be calculated based on the EQ-5D-3L at 90 days and at 12 months after stroke, which consists of the dimensions ‘mobility’, ‘self-care’, ‘usual activities’, ‘pain/discomfort’ and ‘anxiety/depression’ (Dolan, 1997). Participants rate their health on a scale from “no problems” (1) to “extreme problems” (3). By combining the answers, an individual health state out of 243 (3^5) possible health states will be obtained for each participant, with “11111” and “33333” representing the best and worst health state, respectively. These health states will be transformed to the EQ-5D index based on national preference-based value sets with 0 representing death and 1 representing perfect health.

B.2.3. Incremental cost-effectiveness ratio (ICER)

The Incremental Cost-Effectivness Ratio (ICER) will be calculated. It compares costs (€) and effectivness (QALYs) of the both treatment options, ET and BMC:

ICER = ($Costs_{ET}$-$Costs_{BMC}$)/ ($Effects_{ET}$-$Effects_{BMC}$)

The ICER is a point estimate and does not account for uncertainty around this estimate. To determine the probability of cost-effectiveness at different levels of willingness to pay (WTP),

cost-effectiveness acceptability curves (CEAC) will be constructed based on distributions of parameter estimates used in the model.

Further details on the implementation and calculations of cost and utilities are describes below.

B.3. Data

B.3.1. Baseline Cohort

mRS scores and age of the initial population will be determined by pending data of the TENSION trial.

B.3.2. mRS scores

Transition probability between different mRS scores will be derived from TENSION for both arms, that is ET and BMC.

B.3.3. Mortality Data

Mortality for the general population will be calculated from age-specific mortality rates. Mortality is adapted by the realtiv risk of death in each mRS state (Table 1). Furthermore, survial of a recurrent stroke is taken into account by risks of RS in the first year and afterwards (Table 1).

B.3.4. Treatment Effect

For the first year treatment effects will be taken form pending data of the TENSION trial. Treatment effects will be realised by different transition probabilities between individual mRS scores in the model. For the following years, the duration and strength of the intervention effects of ET will be estimated based on expert opinion. For cycles without intervention effects for ET, transition probabilities will be equalised between the two intervention arms.

B.3.5. Quality-adjusted life years

QALYs will be determined based on pending data of the TENSION trial using the EQ-5D index. Mean values will be calculated for each mRS Markov state. Differences in treatment effects between the two interventions will be realized through different transition probabilities.

B.3.6. Cost Data

Care costs will be estimated by mRS scores based on pending data of the TENSION trial. The corresponding care costs will be cacluated for each mRS Markov state. Cost differences between the two interventions will be realised through different transition probabilities.

Price Conversion

All costs will be converted to year 2022 Euros using GDP price level indices.

Discounting

An annual discount rate of 3% will be applied to costs and QALYs based on international recommendations and varied in sensitivity analyses according to current guidelines between 0% and 5% (Institut für Qualität und Wirtschaftlichkeit im Gesundheitswesen, 2020).

Table 1: Parameter values for Markov model

Model Parameter	Parameter Value	Range Used for Univariate Sensitivity Analysis	Distribution in Probabilistic Sensitivity Analysis
Transition Probabilities			
90 Days After Initial Ischemic Stroke			
Health state distribution thrombectomy			
mRS 0	To be provided by WP1	To be provided by WP1	To be provided by WP1
mRS 1	To be provided by WP1	To be provided by WP1	To be provided by WP1
mRS 2	To be provided by WP1	To be provided by WP1	To be provided by WP1
mRS 3	To be provided by WP1	To be provided by WP1	To be provided by WP1
mRS 4	To be provided by WP1	To be provided by WP1	To be provided by WP1
mRS 5	To be provided by WP1	To be provided by WP1	To be provided by WP1
mRS 6 / Dead	To be provided by WP1	To be provided by WP1	To be provided by WP1
Best medical care alone			
mRS 0	To be provided by WP1	To be provided by WP1	To be provided by WP1
mRS 1	To be provided by WP1	To be provided by WP1	To be provided by WP1
mRS 2	To be provided by WP1	To be provided by WP1	To be provided by WP1
mRS 3	To be provided by WP1	To be provided by WP1	To be provided by WP1
mRS 4	To be provided by WP1	To be provided by WP1	To be provided by WP1
mRS 5	To be provided by WP1	To be provided by WP1	To be provided by WP1
mRS 6 / Dead	To be provided by WP1	To be provided by WP1	To be provided by WP1
Survival and Mortality			
Survival after the first year			
Annual Age-specific Mortality for Germany [2]	Yearly probability for 65 years onwards	Yearly probability for 55 and 75 years onwards	
Relative Risk of Death [3]			
mRS 0	1.00	(None)	
mRS 1	1.00	(None)	
mRS 2	1.12	0.82 – 1.56 (CI 95%)	Lognormal (0.113, 0.164)
mRS 3	1.66	1.24 – 2.23 (CI 95%)	Lognormal (0.507, 0.150)
mRS 4	1.92	1.41 – 2.61 (CI 95%)	Lognormal (0.652, 0.157)
mRS 5	2.57	1.92 – 3.43 (CI 95%)	Lognormal (0.944, 0.148)
Survival of Recurrent Stroke			
Risk of Recurrent Stroke Year 0 and Year 1 [4]	0.0491	± 50% [5]	
Risk of Recurrent Stroke Year 2 or more [4]	0.0201	± 50% [5]	
Utility Values			
mRS 0	To be provided by WP1	To be provided by WP1	To be provided by WP1
mRS 1	To be provided by WP1	To be provided by WP1	To be provided by WP1
mRS 2	To be provided by WP1	To be provided by WP1	To be provided by WP1
mRS 3	To be provided by WP1	To be provided by WP1	To be provided by WP1
mRS 4	To be provided by WP1	To be provided by WP1	To be provided by WP1
mRS 5	To be provided by WP1	To be provided by WP1	To be provided by WP1
Costs for care			
mRS 0	To be provided by WP1	To be provided by WP1	To be provided by WP1
mRS 1	To be provided by WP1	To be provided by WP1	To be provided by WP1
mRS 2	To be provided by WP1	To be provided by WP1	To be provided by WP1
mRS 3	To be provided by WP1	To be provided by WP1	To be provided by WP1
mRS 4	To be provided by WP1	To be provided by WP1	To be provided by WP1
mRS 5	To be provided by WP1	To be provided by WP1	To be provided by WP1

B.4. Statistical Analyses

B.4.1. Cost-Effectiveness Analyses

Differences in costs and effects between ET and BMC will be estimated by comparing expected values of costs and effects for each branch of the model. Furthermore, incremental cost-effectiveness ratios (ICER) from a health care perspective will be calculated as the ratio of the difference in mean costs and the difference in mean effects between ET and BMC. Most well accepted medical interventions in industrialized countries have an incremental cost-effectiveness ratio below € 50,000 per quality-adjusted life-years saved (Cohen *et al.*, 2008). Although there is no formal consensus about the precise limit of cost-effectiveness in Germany, any ratios below this threshold will be considered to be cost-effective.

As a robustness check, the analyses will be additionally performed for a per-protocol sample. Furthermore, scenario analysis will comprise subgroups by sex (male/female), occlusion site (M1 segment of the middle cerebral artery (MCA), intracranial segment of the distal internal carotid artery (ICA), both M1 and ICA), NIHSS at enrollment (≤ and > 18), time to treatment (>6 and 6-12 hours) and aspect score (3 and 4-5) as secondary outcomes.

B.4.2. Sensitivity Analyses

Univariate sensitivity analyses will test robustness of the model by varying parameters over plausible ranges (Table 1). To account for time preference of consumption, future costs and QALYs will be discounted at annual rates of 0%, 3%, and 5% (Institut für Qualität und Wirtschaftlichkeit im Gesundheitswesen, 2020). Probabilistic sensitivity analysis will be conducted for all model parameters (Table 1). The willingness-to-pay (WTP) threshold will be 50,000 €/QALY gained and hypothetical cohorts will consist of 10 000 individuals. To determine the probability of cost-effectiveness at different levels of willingness to pay (WTP), cost-effectiveness acceptability curves (CEAC) will be constructed based on assumed parameter distributions (Briggs *et al.*, 2002; Hoch *et al.*, 2002).

B.4.3. Software

All decision-analytic calculations will be performed using the software Treage Pro 2004 Suite, Ver. 5.1 (TreeAge Software Inc., Williamstown, MA, USA).

C References

Briggs AH, O'Brien BJ, Blackhouse G. Thinking outside the box: recent advances in the analysis and presentation of uncertainty in cost-effectiveness studies. Annu Rev Public Health 2002; 23: 377-401.

Cohen J, Neumann P, Weinstein M. Does Preventive Care Save Money? Health Economics and the Presidential Candidates. The New England journal of medicine 2008; 358: 661-3.

Dolan P. Modeling valuations for EuroQol health states. Med Care 1997; 35(11): 1095-108.

Hoch JS, Briggs AH, Willan AR. Something old, something new, something borrowed, something blue: a framework for the marriage of health econometrics and cost-effectiveness analysis. Health Econ 2002; 11(5): 415-30.

Institut für Qualität und Wirtschaftlichkeit im Gesundheitswesen. Allgemeine Methoden. Köln: Institut für Qualität und Wirtschaftlichkeit im Gesundheitswesen; 2020.

Medical University of South Carolina. Rankin Scale Test; 2019.
